# Periorbital Necrotizing Fasciitis Mimicking Allergic Oedema: A Diagnostic Pitfall

**DOI:** 10.7759/cureus.116765

**Published:** 2026-09-23

**Authors:** Danai Barpaki, Stergios K Chaloulis, Diamantis Almaliotis, Konstantinos T Tsaousis

**Affiliations:** 1 Department of Ophthalmology, Papageorgiou General Hospital, Thessaloniki, GRC; 2 Department of Ophthalmology, Hippocration General Hospital, Thessaloniki, GRC; 3 Laboratory of Experimental Ophthalmology, Aristotle University of Thessaloniki, Thessaloniki, GRC; 4 Department of Ophthalmology, Volos General Hospital, Volos, GRC

**Keywords:** corticosteroids, diagnostic reasoning, emergency surgical debridement, periorbital infection, periorbital necrotizing fasciitis, strep. pyogenes infections

## Abstract

Periorbital necrotizing fasciitis is a rare, rapidly progressive, and potentially life-threatening infection that may initially resemble benign conditions, resulting in delayed recognition.

We report the case of a 74-year-old woman who presented with progressive palpebral oedema, which she initially attributed to an allergic reaction. Prior to hospital admission, she had received oral prednisolone 32 mg/day for two days. On hospital admission, marked right upper eyelid oedema, induration, purulent discharge, necrotic cutaneous changes, and systemic signs of infection were present. Laboratory evaluation demonstrated leukocytosis and markedly elevated C-reactive protein, while imaging revealed extensive periorbital soft-tissue involvement. Broad-spectrum intravenous antimicrobial therapy was initiated, and blood and tissue cultures subsequently grew *Streptococcus pyogenes*. Repeated surgical debridement resulted in progressive clinical improvement, with near-complete restoration of eyelid function during follow-up.

This case illustrates the diagnostic challenge of early periorbital necrotizing fasciitis and emphasizes the importance of considering this diagnosis when periorbital inflammation progresses rapidly or develops necrotic or systemic features, even when the initial presentation suggests a benign condition.

## Introduction

Necrotizing fasciitis is a rapidly progressive and potentially life-threatening infection involving the fascia and subcutaneous tissues that requires prompt recognition and treatment [1]. Early manifestations of periorbital necrotizing fasciitis, including erythema, oedema, and pain, may resemble more common and less severe conditions such as preseptal cellulitis or allergic reactions [2]. Disease progression may result in necrosis, visual morbidity, systemic toxicity, and death. Periorbital involvement is particularly rare, with a reported incidence of approximately 0.24 per 100,000 population [3].

Necrotizing fasciitis can be classified microbiologically into polymicrobial and monomicrobial forms. Type II disease is predominantly monomicrobial and is commonly associated with group A beta-haemolytic streptococci, including *Streptococcus pyogenes* [3]. *S. pyogenes *remains an important cause of severe invasive skin and soft-tissue infections and may be associated with rapidly progressive disease [4].

We present a case of *S. pyogenes* periorbital necrotizing fasciitis in a 74-year-old woman who initially attributed her symptoms to an allergic reaction and had received oral prednisolone 32 mg/day for two days before hospital admission. This case highlights the diagnostic challenge posed by early periorbital necrotizing fasciitis and the importance of prompt clinical recognition, antimicrobial therapy, and surgical debridement.

## Case presentation

A 74-year-old woman presented with a four-day history of progressive facial and bilateral palpebral oedema, more pronounced in the right upper eyelid. Symptom onset followed gardening and detergent exposure. No preceding trauma, identifiable skin breach, local infectious focus, or known streptococcal exposure was identified. The patient initially attributed the swelling to an allergic reaction. Prior to hospital admission, she had received oral prednisolone 32 mg/day for two days. Fever developed one day before admission. Her medical history included hypertension, dyslipidaemia, and anticoagulant use. She had no history of diabetes mellitus, malignancy, or immunosuppressive therapy.

On examination, the right upper eyelid was markedly oedematous, indurated, and tender, with severe pain elicited on palpation, purulent discharge, and necrotic cutaneous changes. Pruritus was absent. The left eyelid showed mild erythema and minimal oedema (Figure 1). Fever and tachycardia were present. Initial examination of the right eye was limited by the marked eyelid oedema, which prevented eyelid opening and assessment of visual acuity. 

**Figure 1:**
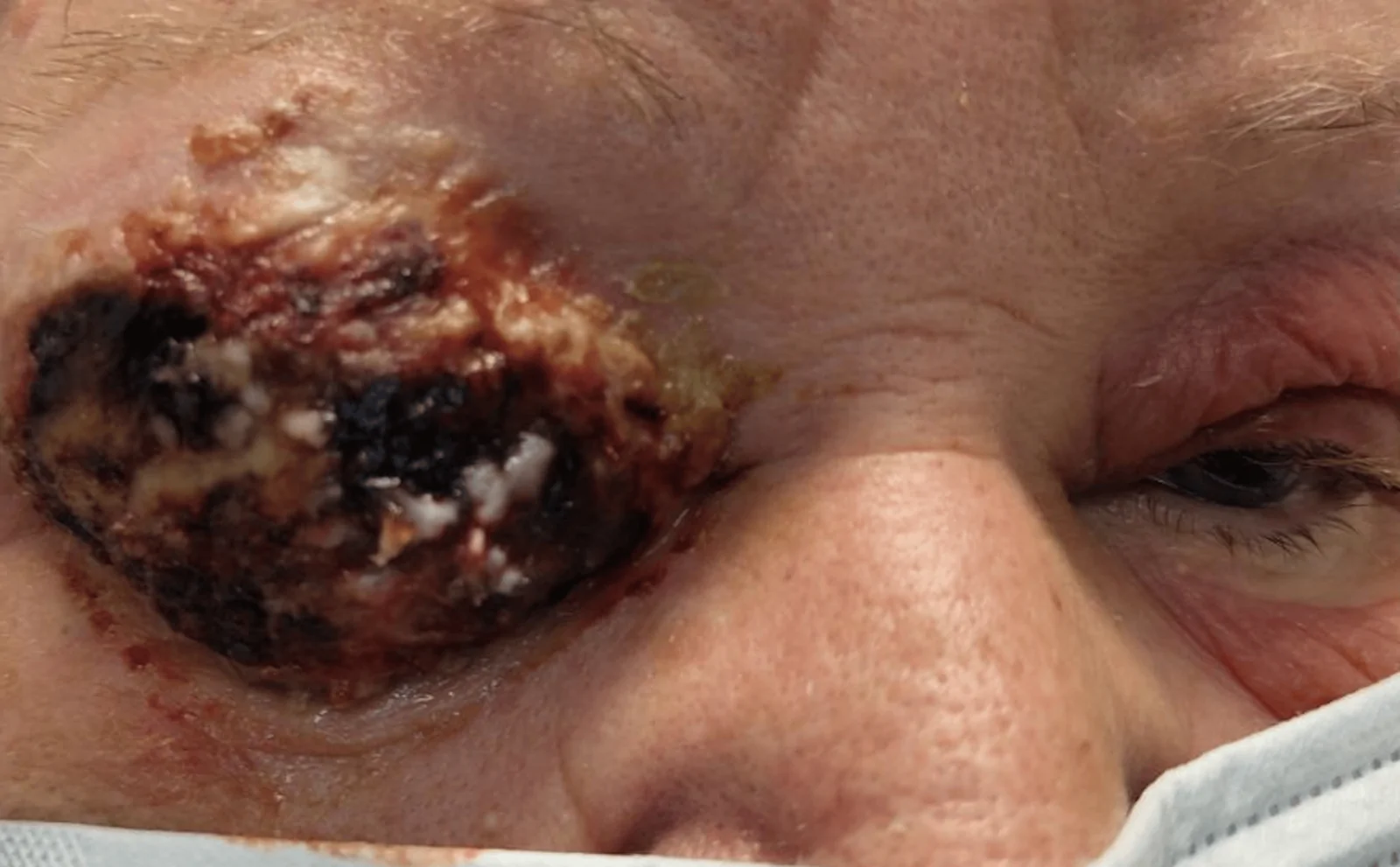
Day 1 of admission Marked, indurated right upper eyelid oedema, with evident cutaneous palpebral necrotic eschar and bloody purulent secretion. Eyelid elevation is impossible, as are visual acuity assessment and further clinical examination of the eye. A mild, erythematous palpebral oedema is noticed in the left eye.

Laboratory tests revealed leukocytosis (24,700/μL, normal range: 4,000-11,000/μL; neutrophils 91.9%, normal range: 50%-70%) and elevated C-reactive protein (278 mg/L, normal range: <5 mg/L). Additional admission values included haemoglobin 13.5 g/dL, serum sodium 141 mEq/L, creatinine 0.9 mg/dL, and glucose 118 mg/dL. The retrospectively calculated Laboratory Risk Indicator for Necrotizing Fasciitis (LRINEC) score was 6 [5]. Orbital CT demonstrated fluid collection anterior to the right globe and extensive periorbital soft-tissue oedema. Empirical intravenous antibiotic therapy with vancomycin and ceftazidime was initiated after obtaining cultures.

Surgical debridement of necrotic tissue was initiated on day two and performed daily. Topical tobramycin and fusidic acid were added. On day three, blood and tissue cultures yielded *Streptococcus pyogenes*, with susceptibility testing demonstrating sensitivity to penicillin, clindamycin, and multiple other antimicrobial agents. As clear clinical improvement had already been observed on the empirical regimen, intravenous vancomycin and ceftazidime were continued, with ongoing clinical and laboratory improvement. Orbital MRI on day four showed no deeper structural involvement.

Inflammatory markers gradually decreased, and systemic symptoms resolved. Figure 2 shows the decrease in oedema and the quantity of necrotic encrustation on day 10, with vague signs of levator muscle function. The left upper eyelid was fully restored.

**Figure 2:**
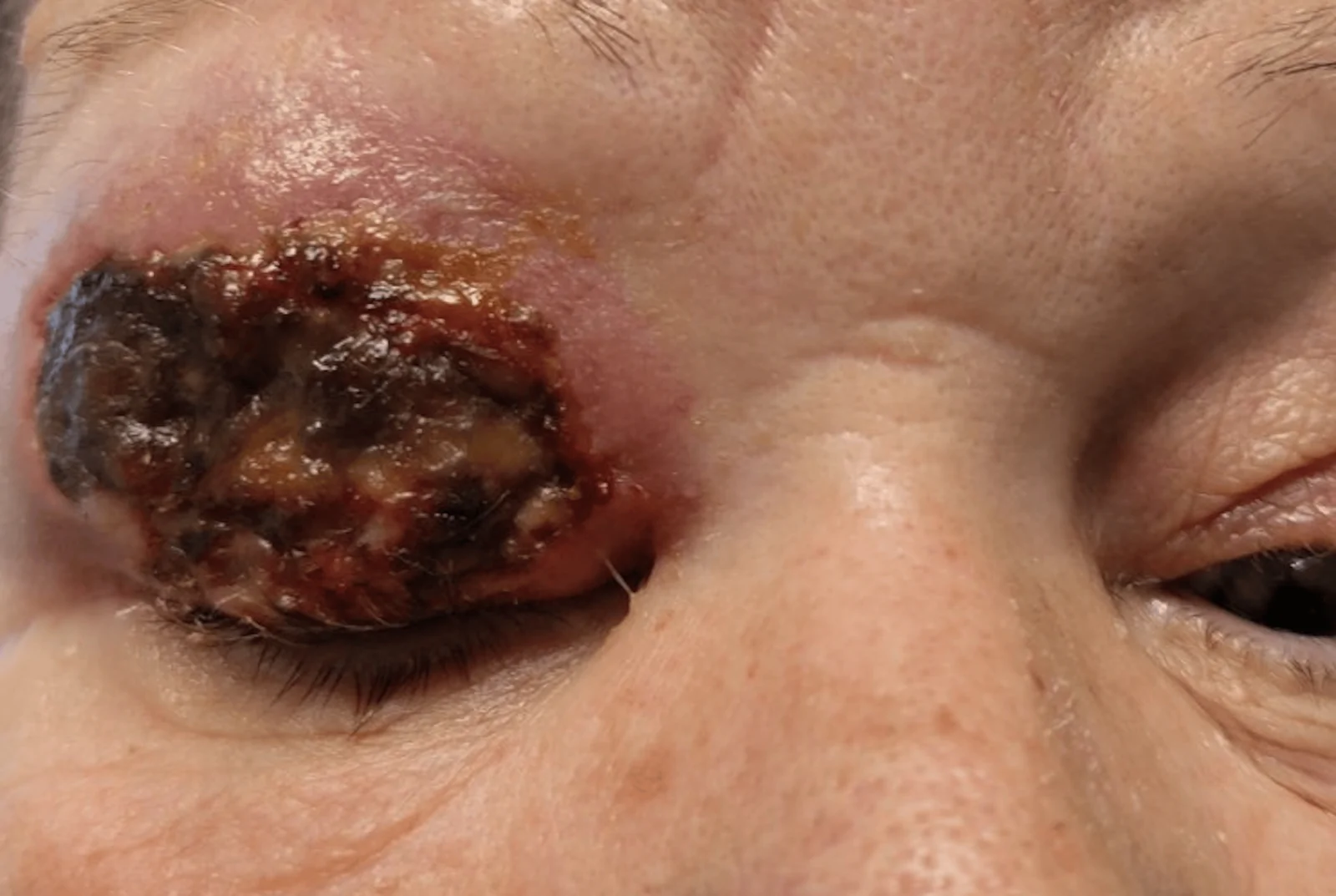
Day 10 of admission Decrease in the oedema and the quantity of necrotic eschars. The levator muscle shows vague signs of function. The left upper eyelid is fully restored.

Debridement continued until day 12, when the patient was discharged. At discharge, the leukocyte count had normalized (9,000/μL), and CRP had significantly decreased. Oral clindamycin and cefaclor were prescribed for seven additional days.

Weekly follow-up, with further debridement performed when needed, demonstrated continued healing with minimal residual changes. Figure 3 shows further improvement in the oedema, subcutaneous tissues, and eyelid function one week after discharge; Figure 4 shows complete resolution of the oedema two weeks after discharge. Once eyelid opening became possible, pupillary responses were preserved, the conjunctiva appeared unaffected, and the cornea was clear. Subsequent examination demonstrated preserved visual acuity and intraocular pressure comparable with those of the contralateral eye. No clinical evidence of lacrimal system involvement was identified.

**Figure 3:**
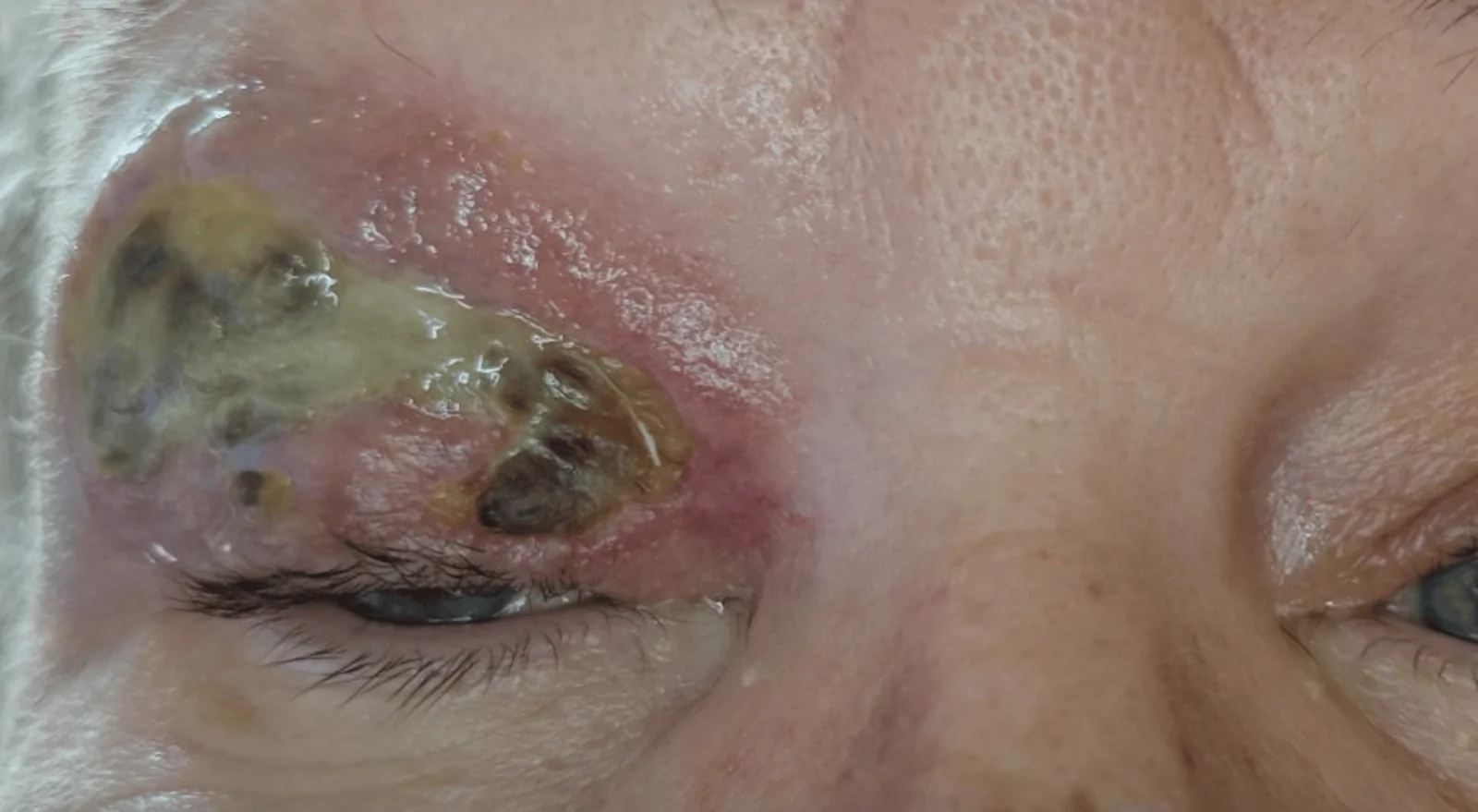
One week after discharge Further improvement of the oedema and subcutaneous tissues. Eyelid opening was evidently improved. The cornea is clear, and there are no signs of bulbar inflammation.

**Figure 4:**
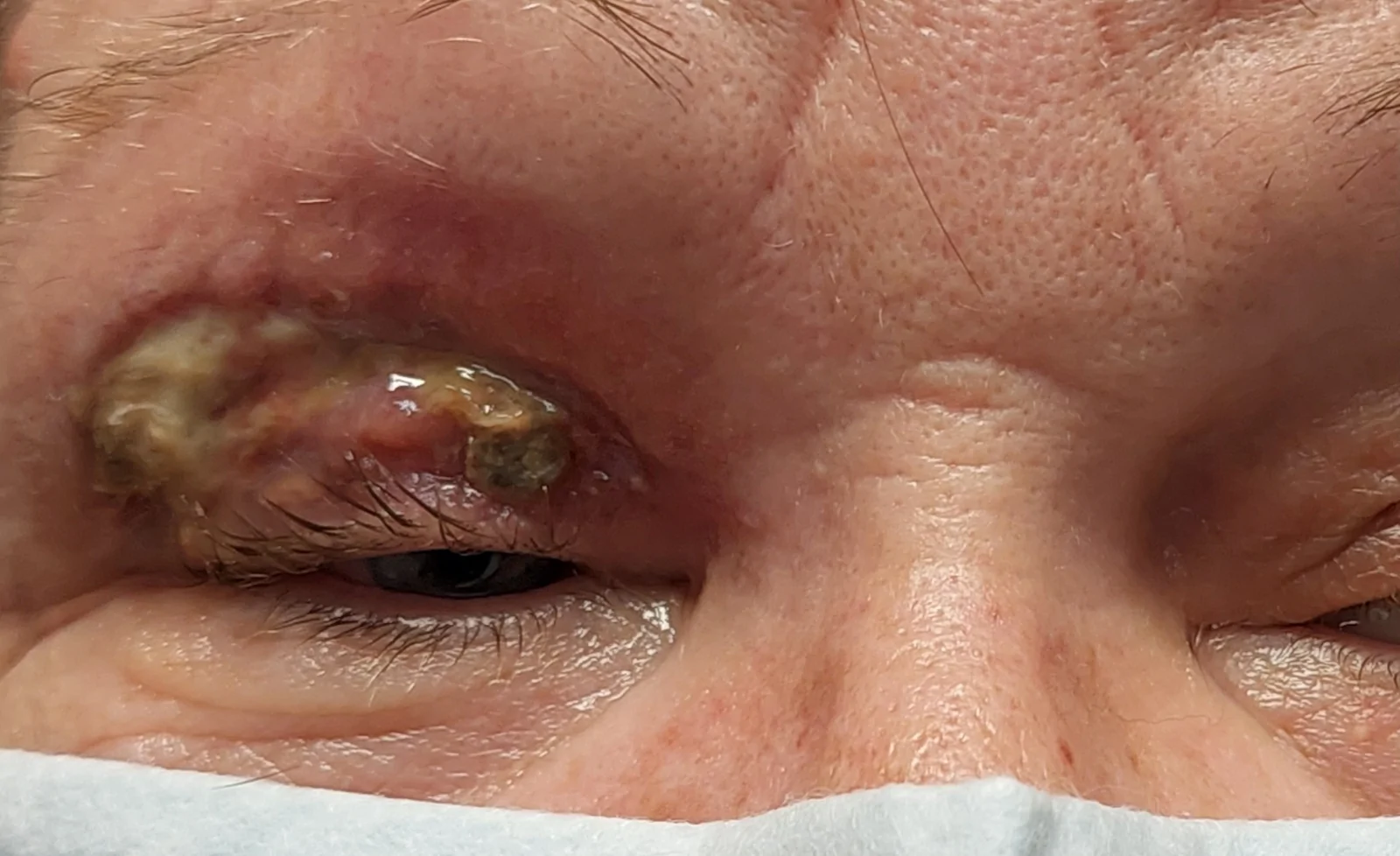
Two weeks after discharge There is no evident oedema of the right upper eyelid. Necrotic tissues are also scarce, and they were debrided. Levator muscle function is almost symmetrical bilaterally.

Finally, there is an evident improvement 48 days after discharge, with only minimal cicatricial skin changes on the upper eyelid (Figure 5).

**Figure 5:**
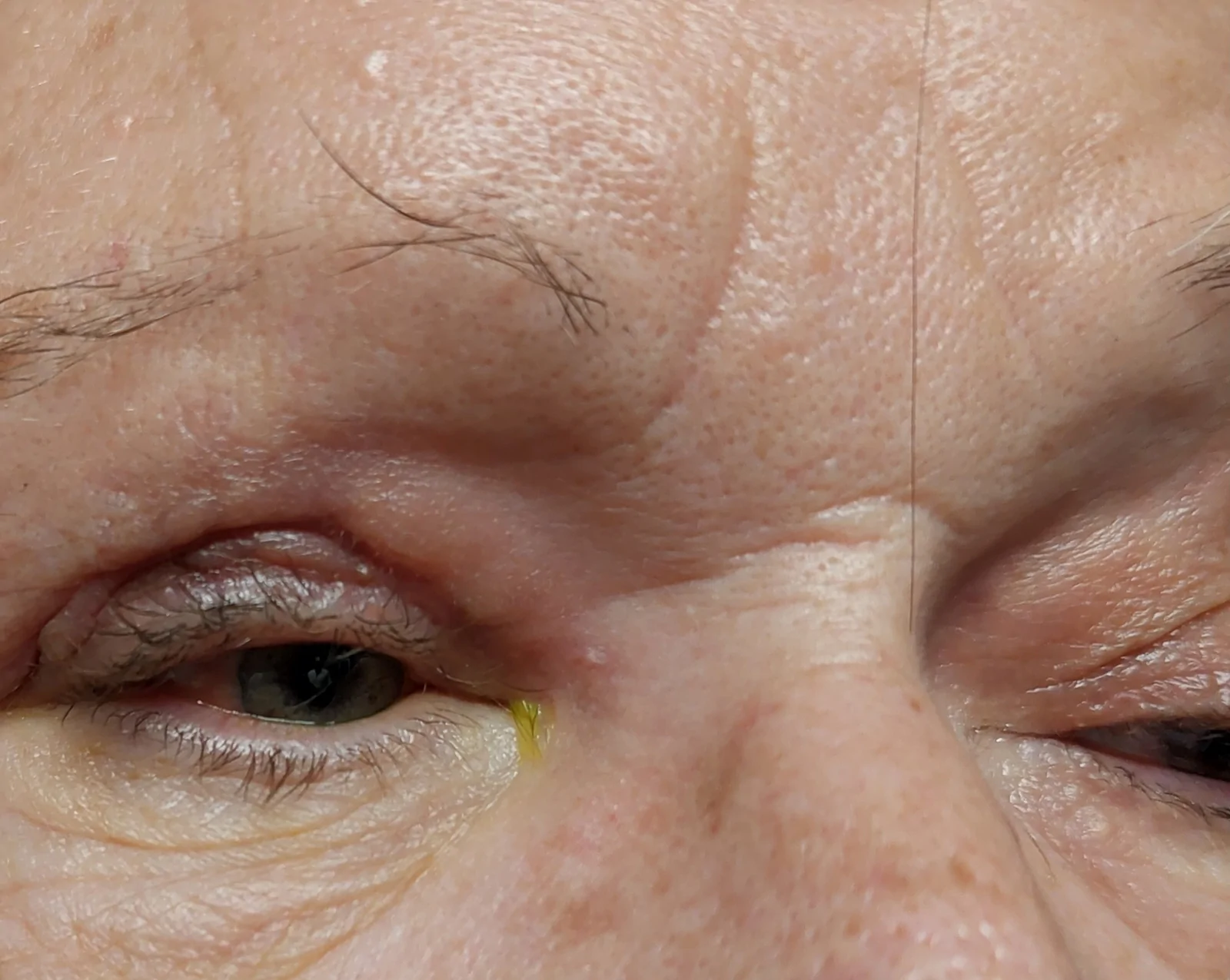
48 days after discharge Some mild cicatricial skin changes are evident. Absence of oedema and necrotic tissue. Complete restoration of levator muscle function.

## Discussion

Periorbital necrotizing fasciitis is uncommon and may initially resemble more benign conditions, including preseptal cellulitis or allergic oedema, making early recognition difficult [2]. Clinical features that should increase suspicion include rapidly progressive oedema and erythema, severe pain or tenderness, induration, skin discolouration or necrosis, and systemic manifestations. This case demonstrates this diagnostic challenge, as the patient initially attributed her symptoms to an allergic reaction and had received oral prednisolone before hospital admission. Although a causal relationship between corticosteroid exposure and disease progression cannot be established from this case, corticosteroid administration in an unrecognized infection may complicate the clinical picture and potentially delay appropriate treatment.

The retrospectively calculated LRINEC score in this patient was 6 [5]. Although the LRINEC score may provide supportive information when necrotizing fasciitis is suspected, it should be interpreted as an adjunct to rather than a substitute for clinical assessment. In this case, the combination of rapid progression, marked induration and tenderness, necrotic cutaneous changes, systemic manifestations, and markedly elevated inflammatory markers prompted urgent treatment despite the diagnostic difficulty of the initial presentation.

Periorbital necrotizing fasciitis may be polymicrobial or monomicrobial. In the present patient, blood and tissue cultures yielded *S. pyogenes*, consistent with type II necrotizing fasciitis [3]. In a systematic review of English-language cases published over a 20-year period, Amrith et al. identified 94 patients from 61 reports. Group A beta-haemolytic Streptococcus was responsible for 51.1% of cases, while toxic shock occurred in 30.9%, visual loss in 13.8%, and death in 8.5% [6]. These findings emphasize that, despite its rarity, periorbital necrotizing fasciitis carries substantial potential for ocular and systemic morbidity and mortality.

Management requires prompt surgical assessment, debridement of necrotic tissue, and appropriate antimicrobial therapy. Broad-spectrum antimicrobial coverage is appropriate initially when the causative organism is unknown, whereas documented group A streptococcal necrotizing fasciitis is generally treated with penicillin combined with clindamycin [7]. In this patient, the empirical vancomycin-ceftazidime regimen was continued after *S. pyogenes *was identified because clear clinical improvement had already occurred; this represents the treatment course followed in this individual case rather than a recommendation for pathogen-directed therapy. Repeated surgical debridement was continued until viable tissue was achieved, accompanied by progressive reduction in inflammatory markers and restoration of eyelid function.

This case highlights several clinical features that may facilitate early recognition of periorbital necrotizing fasciitis, including rapid progression of periorbital inflammation, marked tenderness and induration, development of cutaneous necrosis, and systemic manifestations. The diagnosis should remain a consideration when these features emerge despite an initially benign-appearing presentation.

## Conclusions

Periorbital necrotizing fasciitis should be considered in rapidly progressive eyelid and periorbital inflammation, even when the initial presentation resembles an allergic reaction. Early recognition is particularly important when necrotic changes or systemic signs develop. Prompt broad-spectrum antimicrobial therapy and repeated surgical debridement were associated with a favourable outcome in this patient. This case also underscores the importance of excluding an infectious aetiology before corticosteroids are used for rapidly progressive periorbital inflammation.
